# A New Concept of Sustainable Wind Turbine Blades: Bio-Inspired Design with Engineered Adhesives

**DOI:** 10.3390/biomimetics8060448

**Published:** 2023-09-22

**Authors:** Leon Mishnaevsky, Mohsen Jafarpour, Johanna Krüger, Stanislav N. Gorb

**Affiliations:** 1 Department of Wind and Energy Systems, Technical University of Denmark, DK-4000 Roskilde, Denmark; 2 Zoological Institute, Kiel University, 24118 Kiel, Germany; mjafarpour@zoologie.uni-kiel.de (M.J.); johannakrueger96@gmail.com (J.K.); sgorb@zoologie.uni-kiel.de (S.N.G.)

**Keywords:** wind energy, wind turbine blade, sustainability, lifetime

## Abstract

In this paper, a new concept of extra-durable and sustainable wind turbine blades is presented. The two critical materials science challenges of the development of wind energy now are the necessity to prevent the degradation of wind turbine blades for several decades, and, on the other side, to provide a solution for the recyclability and sustainability of blades. In preliminary studies by DTU Wind, it was demonstrated that practically all typical wind turbine blade degradation mechanisms (e.g., coating detachment, buckling, spar cap/shell adhesive joint degradation, trailing edge failure, etc.) have their roots in interface degradation. The concept presented in this work includes the development of bio-inspired dual-mechanism-based interface adhesives (combining mechanical interlocking of fibers and chemical adhesion), which ensures, on the one side, extra-strong attachment during the operation time, and on the other side, possible adhesive joint separation for re-use of the blade parts. The general approach and physical mechanisms of adhesive strengthening and separation are described.

## 1. Introduction

Large expansion of wind energy is foreseen in Europe in the next years and decades [1]. Europe is expected to install 129 GW of new wind farms over the period 2023–2027, one quarter of which will be offshore. The EU should be building over 30 GW a year on average to meet its 2030 targets. Large and extra-large wind turbines are installed, and the size of blades is increasing. In 2021, the Chinese company MingYang Smart Energy released details of a 264 m tall design with 118 m blades. The Danish firm Vestas developed a 15-megawatt turbine with 115.5 m blades, and Siemens Gamesa Renewable Energy developed a turbine with 108 m blades. Bigger wind turbines allow the capture of more wind and produce more electricity [2].

**Figure 1:**
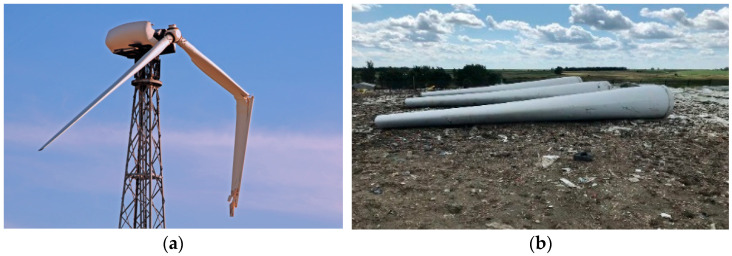
Illustration of two critical problems of wind energy: (**a**) Wind turbine with broken blades, and (**b**) wind blades accumulated in landfill. Photos from Bigstock © collection.

However, recently, an increased failure rate in wind turbines, largely in newer and bigger models, has been reported [3], leading to large expenses for wind turbine manufactures, due to “extraordinary” repairs and upgrades and multimillion losses on rising warranty provisions for GE, Vestas and Siemen Gamesa in 2022. In average, wind turbine blades require repairs every 2 to 3 years, with costs of the order of 30,000 EUR or more per repair [4,5]. Figure 1a shows a damaged wind turbine. The frequency of wind turbine failure offshore can be up to four times higher than for onshore wind turbines [4], while repair costs for offshore wind turbines are also 2 to 2.5 times higher [5]. The frequent and expensive repairs cause higher energy costs and make wind energy less competitive. Thus, the question of how to prevent wind turbine blade degradation and failure becomes especially urgent, due to more and more larger wind turbines installed offshore.

On the other side, another, to some degree inverse, problem becomes critical now: the sustainability and recyclability of wind turbine blades. Currently, wind turbines are recyclable up to 85%. Roughly two thirds of the remaining 15% come from composite materials [6,7]. At end of their life, due to the current blade material, the amount of waste coming from wind turbine blades is expected to reach 43 million tons globally in 2050 [8] (see Figure 1b, showing wind turbine blades accumulated in landfill). Europe will be the first continent to decommission wind turbines and wind farms [8]. In Europe, predictions on the waste amount are 175 kilotons in 2030 and 325 kilotons in 2050 [9]. The solution for this problem includes the development of new recycling (e.g., pyrolysis, solvolysis) technologies, new wind turbine blade materials (like thermoplastic based, vitrimer based, or natural material-based blades) [6], but also the development of disassemblable blades, with detachable parts, which can be reused.

The concept of this paper is as follows. In the preliminary studies [4], it was demonstrated that practically all common blade degradation mechanisms (e.g., coating detachment, blade buckling and collapse, spar cap/shell adhesive joint degradation, trailing edge failure, etc.) are caused by interface degradation, often related to the micro- and nanoscale defects in interfaces. Therefore, the path to prevent the failure and damage of blades lies in the development of methods to strengthen the interfaces in composite blade structures. Further, the development of disassemblable, sustainable blades also requires controlled interfaces, this time, not extra-strong, but rather detachable, separable. Thus, the main assumption of this work is that the structured, engineered interfaces in wind turbine blades are the key to both high durability and sustainability of composite wind turbine blades. This idea is discussed in this paper.

## 2. Concept of Interface Controlled Blade: Lifetime and Recyclability

**Figure 2:**
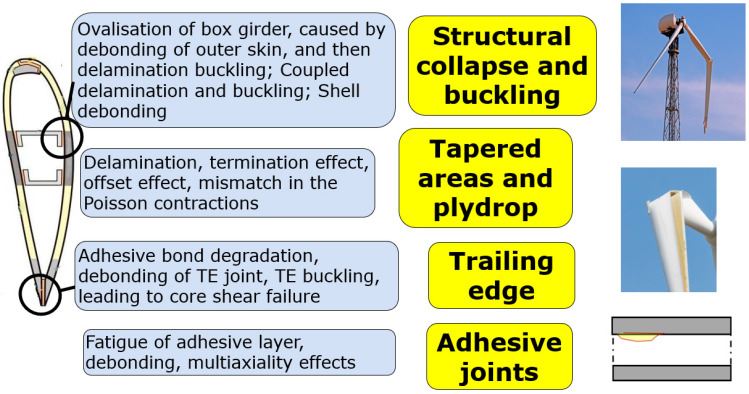
Schema: Damage mechanisms of wind turbine blades. Here, TE = trailing edge.

In order to analyze the mechanisms of damage and failure of wind turbine blades, a number of methods can be applied, including post-mortem analysis of failed blades; full scale testing of blades in laboratories with video-observation and structural health monitoring; analysis of databases and collections of incident reports; direct monitoring of blade deformation and degradation during service (for instance, using non-destructive testing and structural health monitoring methods); testing design of subcomponents (e.g., beam), reproducing parts or elements of the blades (e.g., joints or sandwiches); computational modelling of blade deformation and damage [4]. The review of various studies on the root causes of the failure of wind turbine blades, carried out with the use of these methods, is presented in [4]. Table 1 shows the overview of main mechanisms of wind turbine blade, linking the endangered region of wind turbine blade, observed failure mechanisms and their root causes. Figure 2 shows, schematically, the root causes of the main structural failure mechanisms of the blades. It should be noted that while leading edge erosion and other surface effects (like sizing) are most often observed, and also most expensive damage mechanisms, they are not structural failure mechanisms and are not discussed in the following sections.

An important observation is that virtually all the structural mechanisms of blade failure are controlled by weak, damageable interfaces, causing the buckling and collapse, trailing edge detachment or coating debonding.

## 3. Bio-Inspired Adhesive Layers in Adhesive Joints of Blades

Wind turbines are subjected to long term, permanent randomly varying mechanical and environmental loading, with current usual lifetime of 20 to 30 years. The estimated costs for each repair event are very high, for instance, an average offshore blade repair can cost 30 k EUR, and a new blade can cost 200 k EUR [10].

There are a number of ways to prevent the failure of wind turbine blades, which can be grouped into (a) development of stronger and tougher materials, (b) lighter materials, e.g., carbon composites, which reduce loads on the blades, (c) design solutions, like using transverse cap stiffeners or strengthening trailing edge [4]. However, strengthening of materials is not an infinite process.

In the situation of low and challenging maintenance, extra-long-term service, random varying mechanical and environmental loadings, solutions for the optimization of wind turbine structures can be taken from the biological materials, which are also evolved to bear randomly varying loads over long-term period, without repair option. Looking at strong and tough biological structures (e.g., nacre/shells, skulls/teeth/bones, timber/bamboo) [11], one can observe a quite common feature: these biological composites have extraordinary strength and toughness, which is related with their internal microarchitectures.

These materials at microlevel typically rather often represent layered structures (often, stiff platelets), with nanostructured adhesive layers between stiff platelets). The multiscale design of biomaterials allows the combination of two effects: high stiffness and flexibility, ensured by macroscale structures (e.g., cylindric or cellular wood design, or stiff platelets in nacre) and tough, nanoengineered interfaces, preventing damage even at high deformation.

**Figure 3:**
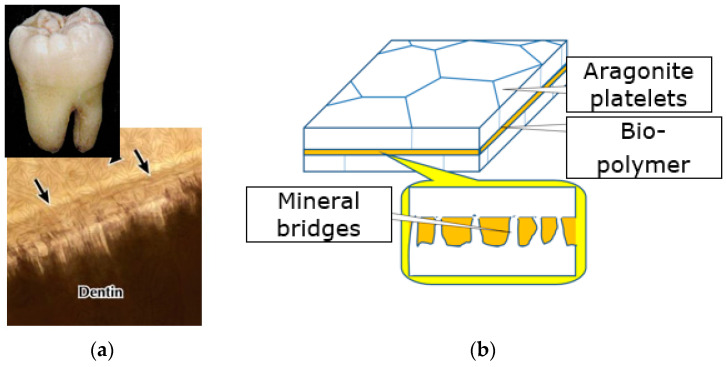
Two examples of extra-strong and tough biological composites, with structured interface layers: (**a**) Tooth, with graded, multilevel dentin-enamel interface, and (**b**) nacre, with biopolymer layer with mineral bridges. The arrows show the dentin-enamel interface region in a tooth.

For instance, nacre, which consists of 95% of CaCO_3_, has a work of fracture 3000 times higher than that of the monolithic CaCO_3_ [11]. Its fracture toughness is controlled by nanostructures in nacre interfaces (i.e., mineral bridges between aragonite platelets in biopolymer layers) [12]. Similarly, graded multi-level dentin-enamel interface in teeth, with scalloped structure (convexities toward dentin, and concavities toward enamel) ensures the extraordinarily high toughness of teeth [11]. Figure 3 shows examples of biological extra-tough structures with nanostructured interfaces, microarchitecture of wood as a layered, fibril reinforced material and nacre, mother of pearl, aragonite platelets, biopolymer layer and mineral bridges.

As applied to composite wind turbine blades, this suggests nanostructuring of adhesive joints and interfaces in blades, mimicking the complex structure of biological adhesives and preventing the majority of blade degradation mechanisms.

## 4. Combined Mechanisms of Adhesion: Mechanical Interlocking and Chemical Adhesion

In a series of works, structural bioinspired adhesives have been developed [11,12,13,14,15,16,17], which exhibit smart adhesion capabilities, like controllable adhesion strength, active adhesion control, reversible adhesion to diverse dry and wet surfaces. So, hydrogel-based, reversible adhesives combining the benefits of both liquid and dry adhesives were developed by Cho et al. [18]. Gecko-inspired hexagonal and lamellae patterned adhesives were developed in [19]. In [20], the authors studied so-called probabilistic fasteners, with two surfaces covered with cuticular micro-outgrowths, ensuring quite reliable and reversible attachment (“probabilistic” because the outgrowths on the contacting surfaces do not correspond exactly to each other, and the interlocking takes place without any precise positioning). Such structures are observed in the wing-locking mechanism in beetles and the head arrester in dragon flies. Further, various types of micro- and nano-hierarchical structures of adhesives (e.g., lamellae, setae, branches and spatulae, self-similar pillar structure, etc.) have been investigated [17].

High potential of such structures for the development of new adhesives was demonstrated. However, structured adhesives and also probabilistic fasteners are developed in most cases for relatively low loads and detachable adhesion.

In this section, the possibility of the development of bio-inspired structured adhesives for high load structures is considered.

**Figure 4:**
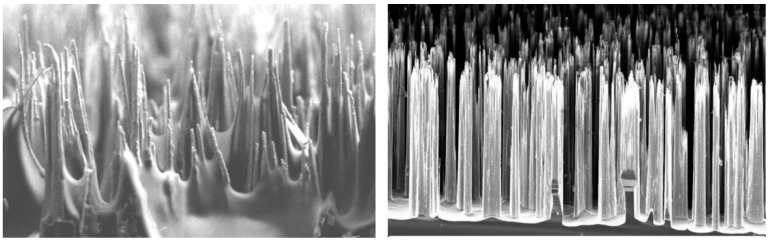
High aspect ratio nanograss, fibrous structures of interfaces (Photo Nicolai Frost-Jensen Johansen, DTU).

**Figure 5:**
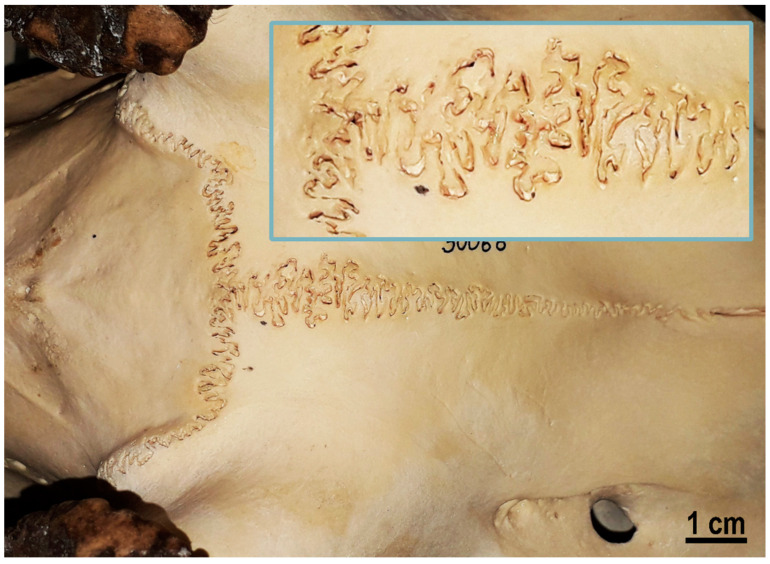
Frontal skull sutures of the male red deer (*Cervus elaphus*). These sutures must withstand very high stresses during male contests. Inset: detailed view.

The idea here is to use a combination of the mechanical and chemical attachment, similar to the adhesive layers in nacre, from polymer and mineral bridges. The adhesion is achieved using the mechanical interlocking of fibrous structures from each side (formed as fibrous nanograss-like, as shown in Figure 4, or brush-like surface structures on sides, or as already mentioned probabilistic fasteners [20]) and is supported via chemical gluing. An example of such mechanically interlocked and also chemically glued biological structure is the red deer skull. Figure 5 shows the frontal skull sutures of a male red deer (*Cervus elaphus*). These sutures must withstand very high stresses during male contests. The insert on Figure 5 shows the mechanically interlocked structures, fibrous joints between the cranial bones and the irregular, but interlocking edges of the bones attached. Sutures provide locomotive shock absorption, to protect the brain [21,22].

## 5. Toughening Potential of Combined Mechanical/Chemical Adhesives: Demonstration

**Figure 6:**
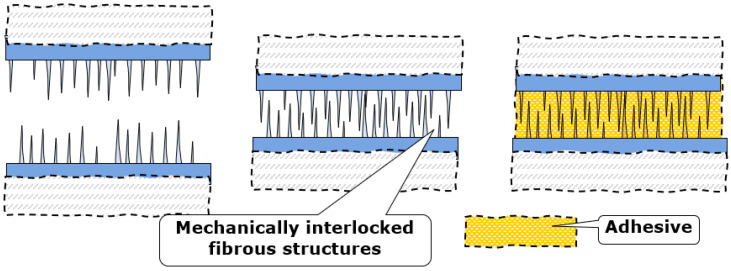
Schema of the combination of mechanical interlocking and adhesive attachment.

Figure 6 shows the schema of the combination of dual attachment (mechanical and chemical), with mechanically interlocked fibrous structures and glue matrix. While fibrous adhesives are often based on soft attachment mechanisms, like friction between fibers or hydrogen bonds, the strength of combined interlocking-chemical adhesives is based on mechanical effects, in particular, reinforcing effect and also toughening mechanism, fiber bridging of defects.

In order to estimate the effect of the fibrous surfaces on the mechanical properties of the adhesive layer, the simple rule of mixture model was applied. Representing the fibers as vertical cylinders, one can estimate that the shear modulus of the polymer adhesive increases using:

(1) $$\alpha =\frac{G_{reinf}}{G_{m}}=\frac{v_{f}G_{f}{+v}_{m}G_{m}}{G_{m}}$$

where v—content of fibers or polymer matrix; indices “f” and “m” mean fibers or matrix, respectively; G—shear modulus; “reinf”—index for reinforced material.

Assuming that the shear modulus of the adhesive is increased by α times, and using the formula by Rosen for shear buckling [23], one observes that such increase in the shear modulus of adhesive leads to the proportional increase in the buckling stress:

(2) $$\sigma \sim \alpha /(1-v_{f})$$

Thus, the combination of fibrous and chemical interface joining increases the material properties of adhesives and reduces the likelihood of the blade buckling.

**Figure 7:**
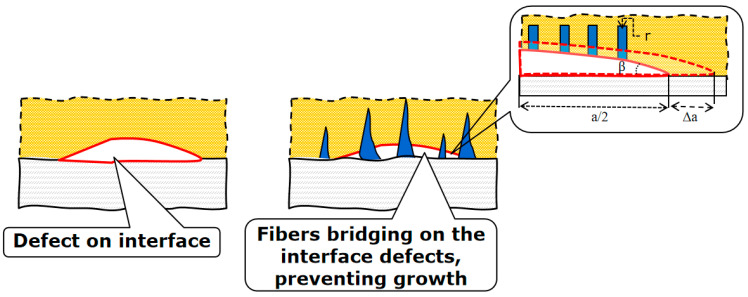
Schema of glued interface with defect (**left**), versus glued and fibrous interface with defect (**right**). Fiber bridging prevents defect growth.

The engineered internal structures of the new adhesives will realize the local damage prevention mechanisms, local stress redistribution (more homogeneous stress distribution instead of high stress concentration, thanks to fibrous surfaces), and the damage tolerance mechanism (when the nanofibers or nanograss prevent the crack growth even when defects are formed, for instance, by so-called “fiber bridging” mechanism). Figure 7 shows the region of adhesive adjacent to the laminate, with a plane defect.

In order to estimate the effect of the fibers available on interfaces on the energy balance of defect growth, we consider the fibers as vertical cylinders, with radius r and N fibers per unit area. The defect is considered a disc-shaped crack, with radius a and angle β at the tip. Assuming the defect extension by small value Δa, the surface of the unbridged crack increases from $\pi {\left(2a\right)}^{2}$ to $\pi {\left(2a+∆a\right)}^{2}$ and the new surface is:

(3) ∆S=π∆a(2a+∆a)

where Δ*S*—new formed surface; Δ*a*—defect extension; and *a*—radius of disc-shaped crack.

However, in the case that the crack is bridged by fibers, which are attached chemically to the polymer, and the crack extension causes detachment of fibers, the additional surface is 2πrh, where h–the length of fiber part which is detached from the polymer; h = Δ*a* tan β. Assuming that the extension of crack detaches only fibers located on one half of the disc-shaped crack, one can estimate the additional created surface as:

(4) $$∆S=2\pi r∆a\;N\tan\beta \frac{\pi a^{2}}{2}=9.87rN∆a\;a^{2}\tan\beta$$

As mentioned above, the value *N* is given in unit per unit area. For the crack angle 5 to 30 degrees, the value tanβ varies from 0.1 to 0.55. If one takes the new surface energy as constant value both for crack growth and fiber detachment, the fracture energy increases due to the fibrous surface, as compared to only polymer adhesive, using:

(5) $$\eta =1+9.87\frac{rN∆a\;a^{2}\tan\beta }{\pi ∆a(2a+∆a)}=1+\frac{\pi rN\;a^{2}\tan\beta }{2a+∆a}$$

For crack angle 10 degrees, Δ*a* = 0.1 a, *a* = 1 mm, *N* = 1000/mm^2^, *r* = 0.001 mm, and *η* = 1.25. Thus, by adding the fibrous mechanism to the adhesion of surfaces, the quite high increase in the adhesive toughness can be achieved.

**Figure 8:**
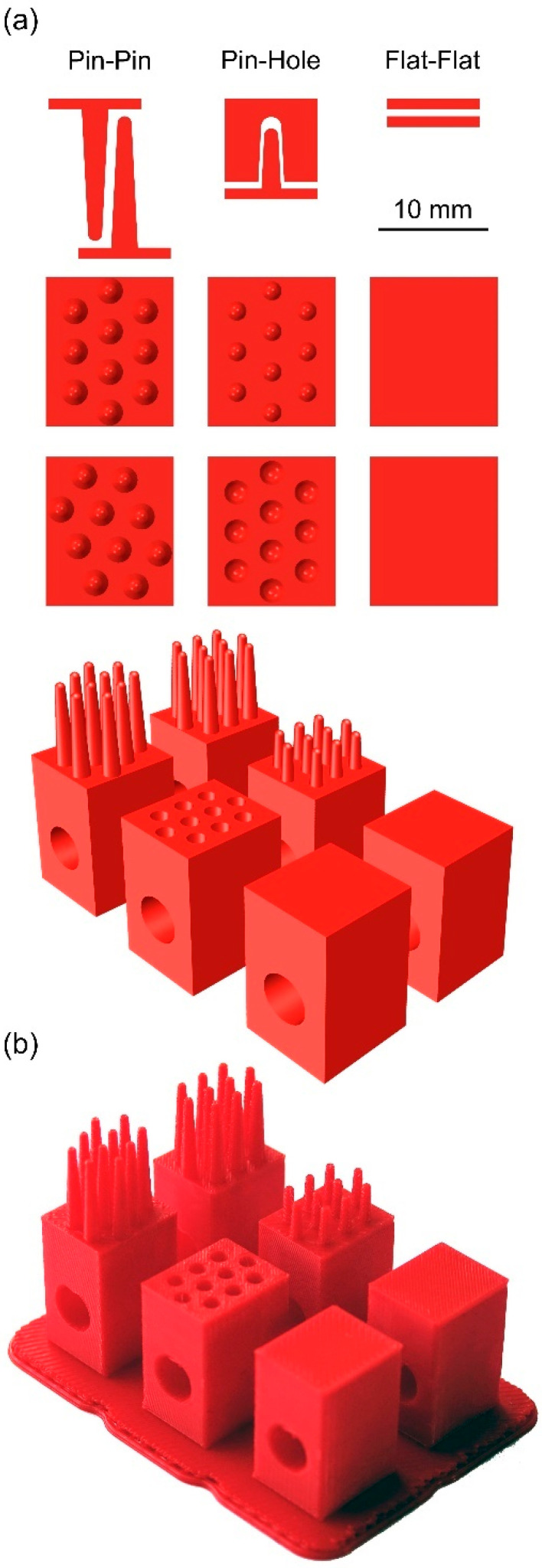
Modeling and manufacturing of the samples with various surface structures. (**a**) 2D schemas and 3D models of adjoining cubes with different bonding shapes, including long pins on the bonding surface (pin–pin), short pins and matching holes (pin–hole), and flat bonding surfaces (flat–flat). (**b**) 3D printed models.

In order to demonstrate the idea, test samples with probabilistic fastener structures were 3D printed and tested. Abaqus software v. 6.14 (SIMULIA, Johnston, RI, USA) was used to develop the 3D models of bonded cubes with different adhesive structures: (i) two cubes with long pins on the bonding surfaces (pin-pin), (ii) a cube with short pins and a cube with matching holes (pin-hole), and (iii) cubes with flat bonding surfaces (flat-flat). While the arrangement of pins in the pin–pin model results in the high friction between the pins and a tight fit, two adjoining cubes in the pin-hole model fit together with a loose fit. The dimensions of the cubes are 12 × 14 × 20 mm, the radius of pins is about 0.5 mm at the base, and the radius of holes defined on the cubes is around 0.6 mm. Figure 8a illustrates the two- and three-dimensional (2D and 3D) views of the developed models. A fused deposition modeling (FDM) 3D printer (Raise3D E2, Raise3D, Irvine, CA, USA) and a polylactic acid (PLA) filament (Raise3D, Irvine, CA, USA) were used to manufacture the developed models (Figure 8b). The printing settings are given in Table 2.

**Figure 9:**
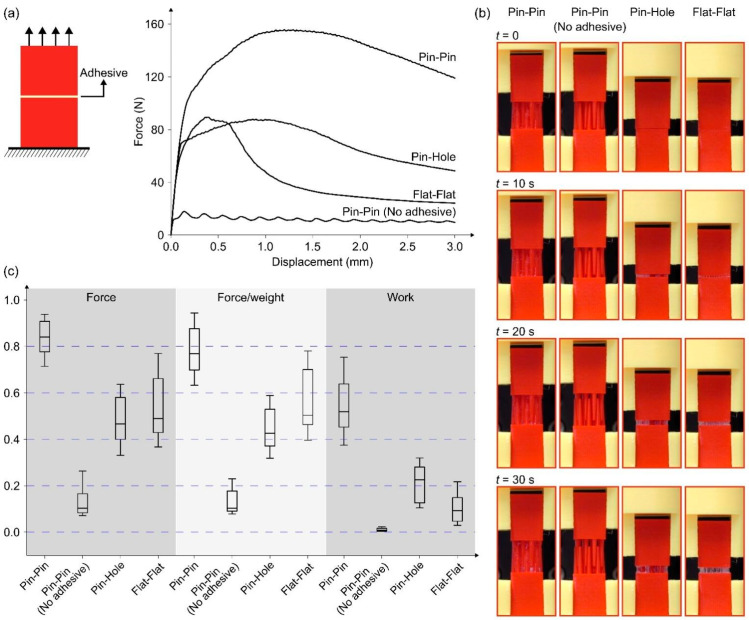
Testing results for the samples with four different bonding shapes. (**a**) Force-displacement curves, (**b**) stages of detachment, and (**c**) the boxplot comparing the maximum force, work, and force divided by the weights of the models.

Using the UHU polyvinyl acetate copolymer all-purpose adhesive (UHU GmbH & Co. KG, Bühl/Baden, Germany), the printed cubes were glued to each other. Models with pin–pin bonding shape were first interlocked into each other and then the interlocked pins were glued. After 48 h, the tensile behavior of the adhered cubes was quantified using a ZwickiLine uniaxial testing machine (Zwick Roell, Ulm, Germany) equipped with a 500 N load cell (Xforce P load cell, Zwick Roell). While one cube was fixed, the other one was pulled with the loading rate of 0.1 mm/s (Figure 9, Video S1). For each type of bonding shape (i.e., pin–pin, pin–hole, and flat–flat), 40 samples were prepared and tested in the same situation (*n* = 40). To study the influence of friction in pin–pin models, 20 additional pin–pin samples were tested with no adhesive (*n* = 20).

The results of the experimental tests are available in Figure 9 and Table 3. Four random force-displacement curves related to four types of samples show the different performance of the cubes with different adhesive structures when subjected to tension (Figure 9a). The mechanical behavior of the models was investigated only up to their maximum load-bearing capacity. Besides the maximum force and work, the values of force divided by the weights of the samples were used to compare their behavior. The data were normalized by dividing the values of each comparing parameter (i.e., force, force/weight, and work) by the maximum value of that parameter and the boxplot was plotted (Figure 9c). The average values of the comparing parameters, besides their standard deviations, are presented in Table 3. In all tests conducted in this study, one cube was totally fixed in all directions, and the other cube was displaced vertically without any rotation.

It can be seen from Figure 9 that the pin–pin structure with additional adhesive ensures the largest fracture energy (work of detachment). This confirms the main assumption of this section: adhesive with both mechanical interlocking and chemical adhesion ensures much better attachment, than that with either only glue attachment (current common technology) or even with only interlocking. The pin–pin structure takes advantage of both friction and larger glued surface area to provide us with an efficient attachment mechanism. Friction plays a crucial role by providing resistance to relative motion between the attached parts. The larger glued surface area further enhances the attachment by creating a greater adhesive bond, distributing the load across a larger surface, and improving the overall load-bearing capacity of the structure.

Models with pin–pin bonding shapes were first interlocked with each other, and then the interlocked pins were glued together. A thin layer of glue covers the pins and fills the gaps. Considering the arrangement of the pins, there is some space in the middle of the two interconnected cubes that is not filled with glue. Both the thickness of the adhesive layer and the air trapped in the joints affect the performance of the structure. Future studies should focus on finding solutions that help control these parameters more precisely to increase the consistency and efficiency of the proposed strategy.

Furthermore, the type of glue used for adhesion is a factor that determines the functionality of the developed structures and their failure. In the pin–pin model, the pins act like cantilever beams in contact with each other. The maximum stress occurs at their base and is significantly influenced by the arrangement of the pins and the type of loading. While in this study, structures have been only loaded vertically for attachment and detachment, the risk of damage in pins was low. In pin–pin samples with adhesive as well, separation in the glued areas happened mostly before any damage to the pins. Future studies can investigate various failure modes in the pin–pin models by using different types of glues and adjusting structural parameters.

## 6. Detachable Interfaces of Composite Blades

As noted above, the problem of the end-of-life management of wind turbine blades is a critical problem of wind energy development [6,7]. The wind turbine blades are designed to sustain decades of service time and do not degrade. This means that they are very difficult to recycle (if recycling is considered as a material separation into constituents, or small parts). As discussed in Section 2, the main degradable parts of the blade are interfaces, adhesive layers, and other thin layers (e.g., coatings). The composite parts in the blades are in fact extremely durable and do not degrade sufficiently even after several decades of operation. This suggests that a blade can be disassembled and taken apart, the supposedly most durable parts (composite laminate parts) can be inspected, while the most damageable parts (adhesive layers, coatings) can be reapplied, thus allowing the further use of the blade materials.

**Figure 10:**
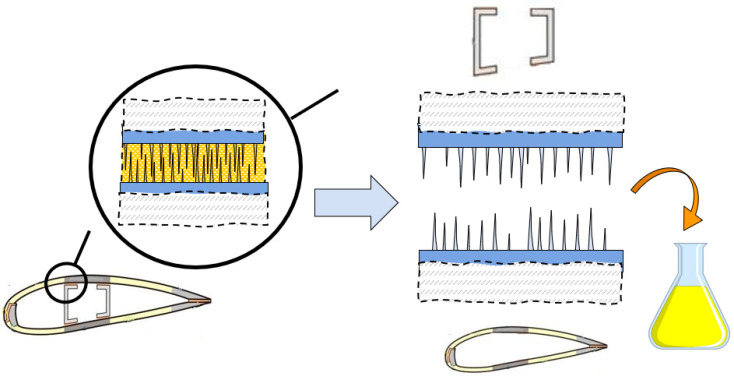
Schema of the separation of a wind turbine blade. After decommissioning with dissolution of adhesive, the fibrous surfaces become detachable allowing to separate the elements of blade structure. The blue arrow shows the transition from the “closed” interface to the debonded interface, with polymer matrix dissolved in yellow flask.

The concept of structured, dual mechanism adhesive layer, presented in Section 4, opens the possibility of the disassemblable, separable blades. Figure 10 shows the schema of such separable adhesive, which includes the dissolution of matrix adhesive, and separation of mechanical interlocking surfaces of blade shall and spar. The matrix adhesive can be decomposed using reactive solvents, for instance, nitric acid, ammonia or glycol, below the critical temperature, or water or ethanol, near the critical temperature [7]. When the polymer matrix part of the adhesive is dissolved, only fibrous interlocking, plus friction remain as attachment mechanisms, and so the parts of a blade can be separated, inspected, and reused in future blades.

Comparing it with the results of previous section (Figure 9 and Table 3), one can see that the removal of adhesive from the pin–pin attached samples allows drastic reduction in the detachment work, almost to zero. Thus, the idea of strong and detachable adhesives is confirmed in the tests above.

## 7. Conclusions

A new concept of extra-durable and sustainable wind turbine blades is presented based on a bio-inspired approach.

The analysis of most often observed damage mechanisms of wind turbine blades lead to the critical role of interfaces and adhesives for the durability and reliability of blades. Most damage mechanisms, e.g., buckling, trailing edge failure, coating detachment, are caused by weaker, damageable interfaces. Given the critical role of interfaces and adhesive layers for the durability of wind turbine blades, the development of extra-tough and separable adhesives should ensure the extended lifetime, reliability, and sustainability of wind turbine blades. The analysis of typical structural biological composites (e.g., nacre, shells) lead to the conclusion that their typical microscale structure includes layered, stiff platelets joined by tough, structured adhesive layers.

In this work, the idea is formulated to use a combination of mechanical and chemical attachment to develop new extra-strong adhesives. The high strength and toughness of new adhesives is achieved using mechanical interlocking of fibrous structures from each side, supported by chemical gluing. It is demonstrated that such adhesives show both higher shear modulus, lower likelihood of buckling, and higher damage tolerance. The potential of bio-inspired structured adhesives, which combine two adhesion mechanisms (i.e., the mechanical interlocking of fibrous surfaces and chemical adhesion) is discussed. The possibility of adhesive joint separation for re-use of blade parts, using two phase adhesives with solvable matrix component is presented.

## Figures and Tables

**Table 1:** Common mechanisms of damage and failure of wind turbine blades.

Endangered Area	Failure Mechanism	Root Causes
Leading edge	Surface erosion, coating debonding	Multiple rain droplet, hail or sand impacts causing surface damage, and/or stress waves, leading to coating debonding
Adhesive joints/bond lines, between the shell and main spar, or between the spar cap and internal stiffeners	Adhesive layers become damaged or debonded, causing buckling or collapse of blade. Blade collapse occurs if the shear web is detached from the shell and buckles. The most often observed failure mechanisms of blades are transverse cracks in the max chord region (initiated as a skin disbond from the sandwich core) and the disbond of the aft shear web from the blade shell in the root-transition zone (triggered by peeling stresses in the adhesive bond lines).	Damage of adhesive joints in the spar area is one of the main processes leading to blade failure. Shear stress is mainly responsible for debonding. Skin debonding from the adhesive joint is the initial mechanism of failure. Apart from buckling-driven failure, two inside surfaces of the sandwich panels can come into contact during the loading, and this has a strong influence on the failure of the trailing edge sections.
Trailing edge	Debonding in the adhesive joint (e.g., due to peeling stress) and/or by buckling of sandwich panels.	Adhesive joints at the trailing edge are other critically important structural parts depending on the reliability of adhesive bond. Blade failure starts from the crack in the bonding at the trailing edge, and then the crack propagates in the laminate.
Tapered areas, plydrop (thickness transitions), root region	Local stress concentration in tapered areas, with thickness transitions, lead to ply delamination (mid spans in the root region, transition from cylinder to an aerofoil, large laminate panels near the maximum chord)	Local stress concentrations, interlaminar stresses in the vicinity of the ply drop-off which can lead to the delamination of plies and the failure of these regions.
Spar and shear webs; buckling	Structural collapse and buckling of blade. Shell debonding from the adhesive joints is the initial failure mechanism, which lead to unstable debonding propagation.	Sudden progressive collapse of a blade is caused by the coupled phenomenon of delamination (interlaminar failure) and buckling, with compressive fiber failure in the delaminated flange material. Local delamination is initiated at the boundary of the adjoining structural elements and in the center of the compressive flange due to local buckling, leading to a sudden structural collapse.

**Table 2:** Three-dimensional printing settings.

3D Printing Settings	
Filament type	Polylactic acid (PLA)
Produced by	Raise3D, Irvine, CA, US
Filament diameter (mm)	1.75
Nozzle diameter (mm)	0.4
Extrusion temperature (°C)	205
Bed temperature (°C)	55
Layer height (mm)	0.2
Fill pattern	Gyroid
Fill density (%)	30

**Table 3:** Results of the experimental tests presented as mean ± standard deviation.

Model	Force (N)	Force/Weight	Work (N × mm)
Pin–Pin	155.7 ± 16.3	31,082 ± 4438	141.3 ± 36.5
Pin–Pin (No adhesive)	24.0 ± 11.6	5117 ± 2044	2.8 ± 1.9
Pin–Hole	89.1 ± 22.3	17,714 ± 3933	54.8 ± 23.3
Flat–Flat	100.0 ± 28.2	22,388 ± 5672	28.1 ± 19.45

## Data Availability

Data are available on request.

## Supplementary Materials

The following supporting information can be downloaded at: https://www.mdpi.com/article/10.3390/biomimetics8060448/s1, Video S1: Mechanical behavior of cubes with different bonding shapes under tension.

## References

[1] (2022). Wind Energy in Europe: 2022 Statistics and the Outlook for 2023–2027.

[2] Frangoul A. (2022). The Race to Roll out ‘Super-Sized’ Wind Turbines Is on. CNBC. https://www.cnbc.com/2022/04/13/green-energy-the-race-to-roll-out-super-sized-wind-turbines-is-on.html.

[3] Williamson R. Wind Turbine Failure Rates Are Rising—Has the Industry Gone Too Big, Too Fast? 9 February 2023, RenewEconomy. https://reneweconomy-com-au.cdn.ampproject.org/c/s/reneweconomy.com.au/wind-turbine-failure-rates-are-rising-has-the-industry-gone-too-big-too-fast/amp/.

[4] Mishnaevsky L. (2022). Root causes and mechanisms of failure of wind turbine blades: Overview. Materials.

[5] Mishnaevsky L., Thomsen K. (2020). Costs of repair of wind turbine blades: Influence of technology aspects. Wind Energy.

[6] Mishnaevsky L. (2023). Recycling of wind turbine blades: Recent developments. Curr. Opin. Green Sustain. Chem..

[7] Mishnaevsky L. (2021). Sustainable end-of-life management of wind turbine blades: Overview of current and coming solutions. Materials.

[8] Liu P., Barlow C.Y. (2017). Wind turbine blade waste in 2050. Waste Manag..

[9] Lichtenegger G., Rentizelas A.A., Trivyza N., Siegl S. (2020). Offshore and onshore wind turbine blade waste material forecast at a regional level in Europe until 2050. Waste Manag..

[10] Mishnaevsky Jr. L., Bendixen B., Mahajan P., Fæster S., Johansen N.F.-J., Paul D., Fraisse A. (2022). Repair of wind turbine blades: Costs and quality. J. Phys. Conf. Ser..

[11] Mishnaevsky L. (2015). Nanostructured interfaces for enhancing mechanical properties of materials: Computational micromechanical studies. Compos. B.

[12] Song F., Soh A.K., Bai Y.L. (2003). Structural and mechanical properties of the organic matrix layers of nacre. Biomaterials.

[13] Heepe L., Xue L., Gorb S.N. (2017). Bio-Inspired Structured Adhesives: Biological Prototypes, Fabrication, Tribological Properties, and Novel Concepts.

[14] Dwivedi P., Singh K., Chaudhary K., Mangal R. (2022). Biomimetic polymer adhesives. ACS Appl. Polym..

[15] Rajabi H., Bazargan P., Pourbabaei A., Eshghi S., Darvizeh A., Gorb S.N., Taylor D., Dirks J.H. (2017). Wing cross veins: An efficient biomechanical strategy to mitigate fatigue failure of insect cuticle. Biomech. Model. Mechanobiol..

[16] Aradhana R., Mohanty S., Nayak S.K. (2018). High performance epoxy nanocomposite adhesive: Effect of nanofillers on adhesive strength and degradation kinetics. Int. J. Adhes. Adhes..

[17] Zhou H., Liu H.Y., Zhou H., Zhang Y., Gao X., Mai Y.W. (2016). On adhesive properties of nano-silica/epoxy bonded single-lap joints. Mater. Des..

[18] Cho H., Wu G., Christopher Jolly J., Fortoul N., He Z., Gao Y., Jagota A., Yang S. (2019). Intrinsically reversible superglues via shape adaptation inspired by snail epiphragm. Proc. Natl. Acad. Sci. USA.

[19] Kaiser I., Tan C., Tan K.T. (2021). Bio-inspired patterned adhesive single-lap joints for CFRP and titanium. Composites B.

[20] Gorb S.N., Popov V.L. (2002). Probabilistic fasteners with parabolic elements:biological system, articial model and theoretical considerations. Philos. Trans. R. Soc. Lond. A.

[21] Sánchez-Villagra M.R. (2010). Suture closure as a paradigm to study late growth in recent and fossil mammals: A case study with giant deer and dwarf deer skulls. J. Vertebr. Paleontol..

[22] White H.E., Goswami A., Tucker A.S. (2021). The Intertwined Evolution and Development of Sutures and Cranial Morphology. Front. Cell Dev. Biol..

[23] Rosen B.W. (1964). Mechanisms of Composite Strengthening, Fiber Composite Materials.

